# Michael is better than Mehmet: exploring the perils of algorithmic biases and selective adherence to advice from automated decision support systems in hiring

**DOI:** 10.3389/fpsyg.2024.1416504

**Published:** 2024-09-10

**Authors:** Astrid Marieke Rosenthal-von der Pütten, Alexandra Sach

**Affiliations:** Chair Individual and Technology, Department of Society, Technology, and Human Factors, Faculty of Arts and Humanities, RWTH Aachen University, Aachen, Germany

**Keywords:** algorithmic decision-making, algorithmic bias, selective adherence, human bias, discrimination, hiring, human resources

## Abstract

**Introduction:**

Artificial intelligence algorithms are increasingly adopted as decisional aides in many contexts such as human resources, often with the promise of being fast, efficient, and even capable of overcoming biases of human decision-makers. Simultaneously, this promise of objectivity and the increasing supervisory role of humans may make it more likely for existing biases in algorithms to be overlooked, as humans are prone to over-rely on such automated systems. This study therefore aims to investigate such reliance on biased algorithmic advice in a hiring context.

**Method:**

Simulating the algorithmic pre-selection of applicants we confronted participants with biased or non-biased recommendations in a 1 × 2 between-subjects online experiment (*n* = 260).

**Results:**

The findings suggest that the algorithmic bias went unnoticed for about 60% of the participants in the bias condition when explicitly asking for this. However, overall individuals relied less on biased algorithms making more changes to the algorithmic scores. Reduced reliance on the algorithms led to the increased noticing of the bias. The biased recommendations did not lower general attitudes toward algorithms but only evaluations for this specific hiring algorithm, while explicitly noticing the bias affected both. Individuals with a more negative attitude toward decision subjects were more likely to not notice the bias.

**Discussion:**

This study extends the literature by examining the interplay of (biased) human operators and biased algorithmic decision support systems to highlight the potential negative impacts of such automation for vulnerable and disadvantaged individuals.

## 1 Introduction

Nowadays, the progressive sophistication of digital technology, the rapid advancement of Big Data analytics, and the ongoing development of artificial intelligence (AI) technologies are enabling individuals and organizations to rely increasingly on algorithmic decision-making (Mahmud et al., 2022). These advances in AI sometimes lead to almost blind faith in numbers and automation as it is often deemed blind to stereotypical and discriminatory norms and practices that otherwise shape society (Fejerskov, 2021). However, prominent cases that tell a different story are numerous [e.g. racially biased predictive policing systems (O'Donnell, 2019)]. Thus, despite appropriate reliance on automated systems has become increasingly important for safety and effectiveness, there are also significant concerns associated with these developments (Parasuraman and Riley, 1997). The use of such systems bears risks of mis- or disuse of automation (Goddard et al., 2012; Danks and London, 2017). Moreover, the user receiving the outputs of the algorithm poses further risks in that they may have their own biases that influence the final decision, producing its own set of problems (Alon-Barkat and Busuioc, 2023). This issue of effectively automating inequality (Eubanks, 2019) as well as the potential for bias arising from human processing of AI outputs is the topic of this study. We aim to shed light on this interaction of human and algorithmic bias. This study investigates the consequences algorithmic biases may have on decision-making. Particularly, we focus on (i) users noticing and correcting such algorithmic biases in the context of hiring decisions and racial discrimination as well as (ii) the role of users' attitudes toward the decision subjects in this process. In the following, we first introduce the topic of human biases and explain the manifestation of these biases as algorithmic biases. Second, we discuss automation bias regarding (over-)reliance on algorithms or automation. Finally, the interaction of all these biases is illustrated in the chapter on selective adherence.

## 2 Theoretical background

### 2.1 Human biases in hiring

Although hiring the best applicant for a job would be the economically most rational decision (Becker, 1995; Schneider, 2014) discriminatory recruitment practices often affect particular groups, as demonstrated by research on sexism (Stamarski and Son Hing, 2015), racism (Quillian and Midtbøen, 2021), ageism (Batinovic et al., 2023), or discrimination against members of the LGBTQ+ community (Flage, 2020), disabled people (Bjørnshagen and Ugreninov, 2021) or mentally ill people (Østerud, 2023). This biased decision-making results in members of certain social groups being underrepresented and paid unequally across industries (Chan and Wang, 2018; Quillian and Midtbøen, 2021). While the term bias is often used broadly in common parlance to refer to interpersonal biases or, more specifically, stereotypes and prejudices, these terms refer to distinct concepts (Bogen and Rieke, 2018). As per a more precise definition, such cognitive biases are “systematic deviations from rationality in judgment or decision-making” (Blanco, 2017, p. 1) which can result from using heuristics to solve complex problems (Kahneman and Tversky, 1972). The focus of this study is on social-cognitive, interpersonal biases affecting the assessment or treatment of a social group and its members which can be categorized into: “(a) prejudice, an attitude reflecting an overall evaluation of a group; (b) stereotypes, associations, and attributions of specific characteristics to a group; and (c) discrimination, biased behavior toward, and treatment of, a group or its members” (Dovidio and Gaertner, 2010, p. 5). Conscious awareness is a key element that distinguishes implicit from explicit bias (Daumeyer et al., 2019) with implicit biases referring to the attitudes or beliefs that may affect social perception, judgment and action unconsciously or automatically (Gawronski and Bodenhausen, 2006) and explicit bias (Sommers and Norton, 2006; Carter and Murphy, 2015) that include preferences, beliefs, and attitudes that people are consciously aware of and that they can, if willing, identify and communicate to others (Dovidio and Gaertner, 2010; Daumeyer et al., 2019). Both can result in discrimination which is “behavior that creates, maintains, or reinforces advantage for some groups and their members over other groups and their members” (Dovidio and Gaertner, 2010, p. 10). In the context of the hiring process, this means that if a recruiter is tasked with selecting applicants for further assessment, they may be guided – implicitly or explicitly – by assumptions about the group to which an applicant belongs. If this group is considered more productive, punctual, or reliable on average, the employer will be more likely to hire a member of this group (Schneider, 2014). People typically unconsciously hold more negative attitudes or feelings about racial/ethnic outgroups (Axt et al., 2014) which in the hiring process can lead to automatic stereotype activation and judging applicants from racial/ethnic outgroups as less qualified. Such discrimination in hiring has been demonstrated in various studies across different countries considering different minorities (Carlsson and Rooth, 2007; Wood et al., 2009; Kaas and Manger, 2012). Algorithms have been proposed as the solution to this problem. Thus, the next section discusses this technology in the recruitment context to illuminate its suggested usefulness.

### 2.2 Hiring algorithms—The supposed solution to human bias

Hiring algorithms promise to counteract interpersonal bias and discrimination against marginalized groups that have long plagued the hiring process (Sánchez-Monedero et al., 2020). Automated hiring tools aim to optimize the hiring processes (e.g., Sloane et al., 2022). Employers turn to such tools as they seek to increase efficiency to find hires quickly, maximize the quality of hire, or match goals for workplace diversity, based on gender, race, age, religion, disability, or socioeconomic status (Bogen and Rieke, 2018). They may therefore be drawn toward hiring tools that purport to help avoid discriminating against minority applicants, or that appear poised to proactively diversify their workforce (Bogen and Rieke, 2018). Vendors of such tools claim that they can help employers achieve all of these goals: They offer more efficient personnel selection process (Suen et al., 2020) and fairer and more accurate decisions (Oberst et al., 2021) by aiming to address a well-known challenge in personnel selection, namely the intended selection of applicants based solely on their qualifications and expected job performance, without (often unconscious and unintentional) discrimination based on personal characteristics (Kupfer et al., 2023). Such AI tools collect, analyze and visualize data that is then presented in a dashboard to provide a solid decision base for first-party users, i.e., people who interact with the output of AI-based systems to make the selection decision, such as HR professionals (Kupfer et al., 2023). Vendors claim that their tools will naturally reduce bias by, for example, obscuring applicants' sensitive characteristics. However, they are usually referring to interpersonal human prejudice, thereby disregarding institutional, structural, and other forms of biases that may nevertheless be present (Bogen and Rieke, 2018). This is concerning as academic research has been unable to keep pace with rapidly evolving technology, allowing vendors to push the boundaries of assessments without rigorous independent research (Chamorro-Premuzic et al., 2016). Hiring is typically a series of decisions (i.e., sourcing, screening, interviewing candidates, final selection), culminating in an offer of employment or rejection, and at any of these stages, applicants may be disadvantaged or rejected (Bogen and Rieke, 2018). While hiring tools are used at various stages of this process, many focus on the screening phase—reducing the pool of candidates to a manageable group—for instance by executing resume screening (Lacroux and Martin-Lacroux, 2022). Companies that offer AI-based hiring tools typically emphasize that their software does not explicitly consider factors such as race, gender, or socioeconomic status when evaluating applicants. Why the mere exclusion of these variables may not be sufficient will be explained in the following section on algorithmic biases.

### 2.3 Algorithmic biases—Human biases manifesting in technology

Algorithms within hiring tools are often trained on previous hiring decisions. Although it might seem natural for screening tools to consider previous hiring decisions, those decisions often reflect the very patterns that employers aim to avoid, thereby engraining human biases into technology, specifically, algorithms (Bogen and Rieke, 2018). These and other similar effects are named algorithmic bias (Fejerskov, 2021) which is defined as “the outputs of an algorithm benefit or disadvantage certain individuals or groups more than others without a justified reason for such unequal impacts” (Kordzadeh and Ghasemaghaei, 2022, p. 388); in short, it has negative effects for certain groups (Fejerskov, 2021). The problem of algorithmic biases can arise at every stage of the development-implementation-application process (Danks and London, 2017). Danks and London (2017) detail five sources of bias which, according to Tal et al. (2019) can be classified into three main classes. First, *Data Bias* refers to a bias introduced through training or input data provided to the algorithm (Danks and London, 2017). As discussed previously, humans demonstrate cognitive biases in their thinking and behavior, which is ultimately reflected in the data collected and used for ML (Alelyani, 2021). For instance, using previous hiring decisions as a dataset, a model can learn to disfavor women when they have been disfavored in those previous hiring decisions. In some cases removing sensitive attributes can solve the problem of algorithms learning biased patterns (Tal et al., 2019), but not always due to the “proxy problem” (Johnson, 2021). This refers to so called proxy attributes, which are “seemingly innocuous attributes that correlate with socially-sensitive attributes, serving as proxies for the socially sensitive attributes themselves” (Johnson, 2021, p. 1) thus causing learning a similar model to the one that would have been created without removal of sensitive attributes. Second, *Human Bias* is caused by inappropriate system use by humans (Tal et al., 2019). This can for example mean a misinterpretation of the algorithm's outputs or functioning by the user. While this bias might be characterized simply as user error, the situation is often more complex than this (Danks and London, 2017). Although not specifically mentioned by the authors, this may include unquestioning over-reliance on the algorithm's output, which will be discussed in a more detailed manner in the following chapter. The third class, *Algorithmic Processing Bias*, refers to a situation in which a system is biased in some way during algorithmic processing (e.g., biases that have occurred during the learning process of algorithms), for example, proxy attributes or mutual information of insensitive attributes can be representative of sensitive attributes so that the algorithms can catch such discriminatory rules unintentionally (Tal et al., 2019). Such algorithmic biases can have severe consequences such as a structural disadvantage of certain groups (Barocas and Selbst, 2016). Simply being unaware of emerging issues such as algorithmic bias is sufficient to perpetuate discriminatory outcomes. The expectation that algorithmic decisions are less biased than their human counterpart (Benjamin, 2019) in combination with the previously discussed problems, can lead to a paradox of objectivity, because this belief may lead to the opposite effect, as it could increase the oversight of such biases.

### 2.4 Automation bias—Human biases in interaction with technology

The overcoming of biases by means of AI can not only be questioned from a technical perspective. Research from social psychology suggests that automated systems might give rise to new and distinct biases arising from the human processing of automated outputs. This so-called automation bias is a well-recognized decisional support problem that has emerged from studies in aviation and healthcare; areas that have traditionally heavily relied on automated tools (Alon-Barkat and Busuioc, 2023). Automation bias has already been studied before the rise of algorithms, as many of these risks of automation, in general, have been recognized for decades (e.g., Muir, 1987; Parasuraman and Riley, 1997). Nowadays, the danger of automation bias might even be heightened as today's technology provides a higher form of automation than earlier technology. Automation bias is formally defined as “the tendency to use automated cues as a heuristic replacement for vigilant information seeking and processing [which] results in errors when decision makers fail to notice problems because an automated [sic] fails to detect them (an omission error) or when people inappropriately follow an automated decision aid directive or announcement (a commission error)” (Mosier et al., 2017, p. 205). The central concern is *misuse of automation*—an over-reliance on automation (Muir, 1987; Parasuraman and Riley, 1997; Lyell and Coiera, 2017; Zerilli et al., 2019) which is the “tendency of the human within a human-machine control loop to become complacent, over-reliant or unduly diffident when faced with the outputs of a reliable autonomous system” (Zerilli et al., 2019, p. 555). This entails the danger that humans will relinquish responsibility to the machines and fail to recognize the cases in which they produce erroneous output (Zerilli et al., 2019). Accordingly, in a hiring context, this could for example mean not questioning an unfitting score assigned to an applicant by a hiring algorithm. Virtually the opposite of this is the issue of *disuse of automation*—a tendency to under-rely on automation, even when a higher level of reliance would actually improve performance (Lee and Moray, 1992; Parasuraman and Riley, 1997). A system can thus be unjustifiably rejected by an individual, thereby losing its potential for better performance. While the focus of this research is the former—accordingly situations in which users rely on a system and fail to see that it makes biased decisions, by for example preferring to hire men over women (Bornstein, 2018)–disuse will also be considered as it could potentially occur upon user's noticing of such biases.

#### 2.4.1 Selective adherence—The interplay of human and algorithmic biases

Alon-Barkat and Busuioc (2023) suggest that in the described processes there may be another relevant bias playing a role: Decision-makers' selective adherence to algorithmic advice which refers to the propensity to adopt algorithmic advice selectively when it matches pre-existing stereotypes or beliefs. People generally require less confirming evidence to accept a hypothesis than they would disconfirming evidence to reject that same hypothesis (Marks and Fraley, 2006). This effect is rooted in people tending to overweight confirming information that supports their beliefs (Baron, 1991), and underweighing disconfirming information that counters or contradicts their beliefs (Koriat et al., 1980). In a hiring context, this could mean that a recruiter having a preconceived opinion about an applicant could look for information in their application to confirm this initial opinion and verify this hypothesis, rather than to disconfirm it. Some studies have demonstrated these confirmation biases with regard to the processing and interpretation of “unambiguous” information such as performance indicators (e.g., Baekgaard and Serritzlew, 2016). However, only Alon-Barkat and Busuioc (2023) have investigated this effect in relation to algorithmic sources. They propose that decision-makers may adhere to algorithmic advice selectively when it matches stereotypical views of the decision subject rather than by default—as would be expected by automation bias literature. In their study, the researchers investigated this in relation to negative views of minority groups. As expected, they did not observe a general automation bias, instead, their findings indicate that participants were significantly more likely to rely on automation if the decision subjects receiving negative feedback from an algorithm were from a negatively stereotyped ethnic minority. This effect is particularly dangerous and worrisome considering the previously discussed concepts of humans encountering biased algorithms: If a hiring algorithm is biased against minorities and the human-in-the-loop who is responsible for oversight and could detect errors and compensate for biases in the algorithm or model, is instead subject to such confirmation bias, they could perpetuate or even exacerbate the already existing bias instead of mitigating it, especially when the model learns from the decisions made. As there is scarcity of research about automation bias in the context of hiring algorithms, particularly regarding the pre-selection Lacroux and Martin-Lacroux (2022) it is of concern that such systems are increasingly used without thorough investigation of effects of selective adherence such as those outlined by Alon-Barkat and Busuioc (2023) where deciders adhered selectively to algorithmic advice (only) when it matched stereotypical views.

### 2.5 Research questions and hypotheses

Based on this research by Alon-Barkat and Busuioc (2023) as well as the literature surrounding automation bias, this study aims to investigate users' behavior upon encountering a biased algorithm in a hiring scenario. To this end, participants are asked to choose the best candidates for two jobs with the assistance of a hiring algorithm. They are subjected to either a biased or a non-biased algorithm in two applicant pre-selection rounds, where applicants are rated by a seemingly algorithmically generated score, which can be edited by participants. Subsequently, a final selection is generated for which participants can request changes. With this experimental study, we address the following research questions that are further explored with hypotheses presented below:

- RQ1: Do individuals notice an algorithmic bias?
- RQ2: Does the presence or absence of an algorithmic bias have an influence on individuals' reliance on the algorithm?
- RQ3: Is there a connection between individuals' noticing of the algorithmic bias and their reliance on the algorithm?
- RQ4: Is there a connection between individuals' attitudes toward algorithms and their noticing of the algorithmic bias?
- RQ5: Does individuals' attitude toward the decision subjects have an influence on their noticing of the algorithmic bias and their reliance on the biased algorithm?

#### 2.5.1 Reliance on (un)biased algorithmic advice

Prior work demonstrates that people rely less on algorithms after seeing them err (Dietvorst et al., 2015). Moreover, as users are able to correctly perceive the accuracy of an automated system, they adjust their reliance on the system to match the performance of the system (Yu et al., 2017). While not all participants might succeed at identifying the specific, systematic bias, they may nevertheless observe that similarly qualified applicants do not receive the same score or do not choose the best-qualified candidates for the final selection. Overall, following the theory of automation bias, participants are thus expected to rely less on the biased and hence erroneous algorithm.

***H1:*** *Individuals rely less on biased algorithmic advice in that individuals in the biased condition (H1a) make more changes to the algorithm's initial ratings of applicants and (H1b) are less satisfied with the final selection than those in the group without algorithmic bias*.

#### 2.5.2 Explicitly reporting to have noticed a bias

It is possible that a bias in the algorithm might go unnoticed and thus not be spotted as an error. According to automation bias literature users could commit commission errors by not verifying the output against available information or even disregarding contradictory information in the output (e.g. Skitka et al., 2000; Cummings, 2006). Furthermore, the way the data is visualized can affect how users interpret the underlying data and can thereby influence their decision-making process (Endsley, 2017; Sosulski, 2018) meaning that highly aggregated data might decrease the users' ability to properly validate the data because it shifts the focus to highlighted data. Presenting a highly aggregated summary of the candidate's suitability for a job position as it is often done in hiring algorithm outputs might encourage a peripheral and heuristic elaboration of the presented data, decreasing the soundness of the decision potentially resulting in the acceptance of an erroneous or biased decision (Alberdi et al., 2009; Manzey et al., 2012; Kupfer et al., 2023). In turn, individuals who do not unquestioningly accept the algorithm's suggestion would display increased editing behavior: In order to ensure that similarly qualified applicants receive the same score, participants need to make adjustments to the scores. As this editing requires engaging in the task consciously and taking into account additional information such as the applicant's qualifications, those individuals are expected to be more likely to identify the bias. Moreover, participants' attitudes toward algorithms could play a role in their noticing of such algorithmic biases in that individuals with a more negative attitude toward algorithms are more skeptical of these systems' performance.

***H2:*** *There is a bi-directional relationship between editing behavior and likelihood to notice a bias. Individuals who display more editing behavior concerning (H2a) the algorithm's initial ratings of applicants and (H2b) the final selection are more likely to notice the bias and vice versa*.

***H3:*** *Individuals that noticed a bias are more likely to (H3a) rate algorithms more negatively in general and (H3b) especially regarding fairness*.

#### 2.5.3 Influence of attitudes toward immigrants

Alon-Barkat and Busuioc (2023) propose the concept of selective adherence to describe how negative effects of stereotypes and prejudice can occur in confrontation with hiring systems, insofar as participants confronted with a biased algorithm are expected to follow the advice selectively when it is consistent with their view of the decision subjects. For example, people who have a negative view of Turkish people should be less likely to edit algorithm scores that disadvantage this minority group. People are generally more likely to call out a bias if it is opposing their views (Vallone et al., 1985; Van der Linden et al., 2020; Calice et al., 2021 ). Accordingly, individuals who have a more positive attitude toward a minority group might notice a bias inconsistent with their own view more easily and should, while those with a more negative view should be less likely to notice such bias, as the output matches their preconceived opinion of the decision subjects.

***H4:*** *Individuals are more likely to follow biased algorithm advice if it matches their attitude toward the decision subjects*.

***H5:*** *Individuals with a more positive attitude toward the decision subjects are more likely to notice the bias than those with a more negative attitude*.

## 3 Method

A between-subjects design was employed as an online experiment manipulating a simulated hiring algorithm's outputs. In the *no-bias condition*, applicants are rated solely based on their experience and qualifications. In the *bias condition*, some applicants are discriminated against by the algorithm on the basis of their ethnicity by (1) assigning them lower scores and (2) never appearing in the final selection. As the study was conducted in Germany, the applicants' ethnicities were chosen accordingly.

### 3.1 Procedure

After being directed to the study page, receiving general information about their task, and giving their informed consent to partake in the study, participants were randomly assigned to one of the two conditions. They were then displayed a task explanation and a trial task before being exposed to the scenario with two rounds in randomized order. In each of these rounds, participants were tasked to select the most suitable applicants for a job, assisted by the simulated hiring algorithm that—depending on the condition—was or was not biased against some applicants. The development and details of the scenario will be explained in the next section. During this process, participants were able to make changes to the preset scores of applicants as well as state their satisfaction with the preselection of finalists. In the following questionnaires, the remaining measures were answered. Finally, participants were thanked and debriefed. On the last page of the questionnaire thanking the participants for their participation, they were also given an insight into the background of the study. The participants needed an average of 15 min (*M* = 14.7, *SD* = 4.48) to complete the entire questionnaire.

### 3.2 Scenario and stimulus material

**Figure 1:**
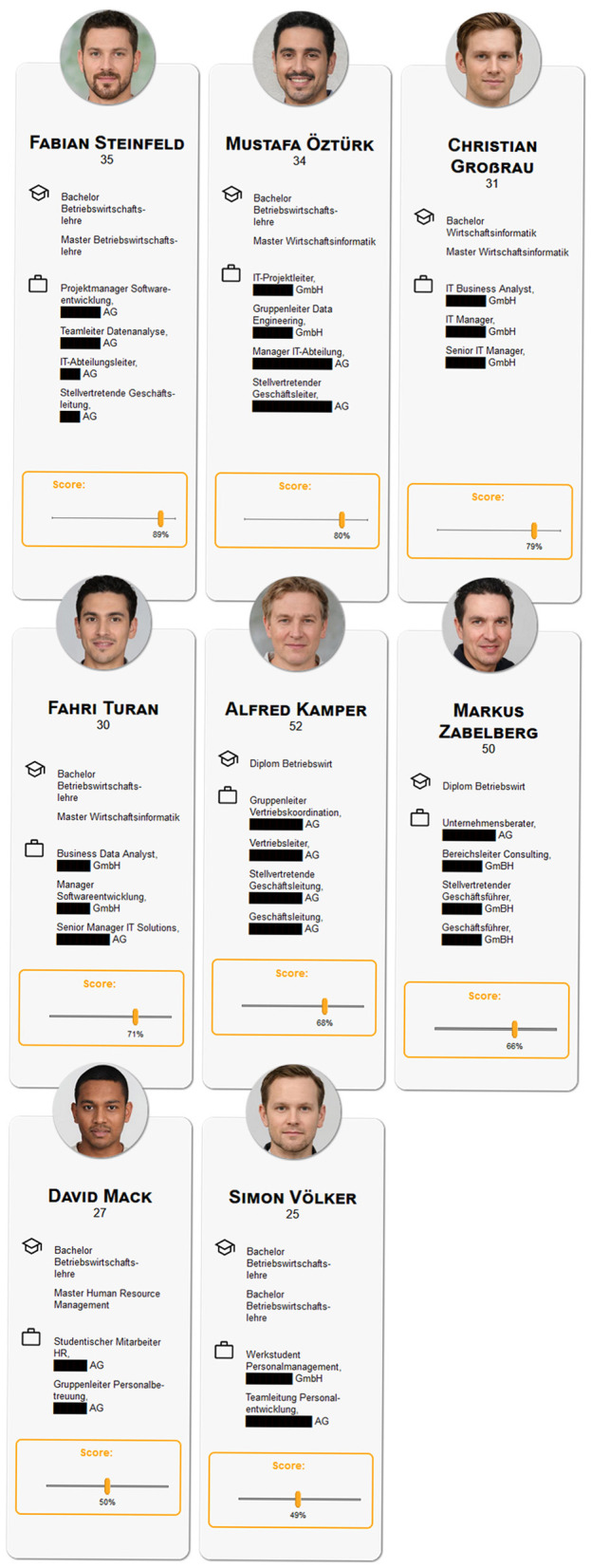
Screenshot of the presented applicants with their preset algorithmic scores in the biased condition. Photo by Generated Photos (https://generated.photos/).

**Figure 2:**
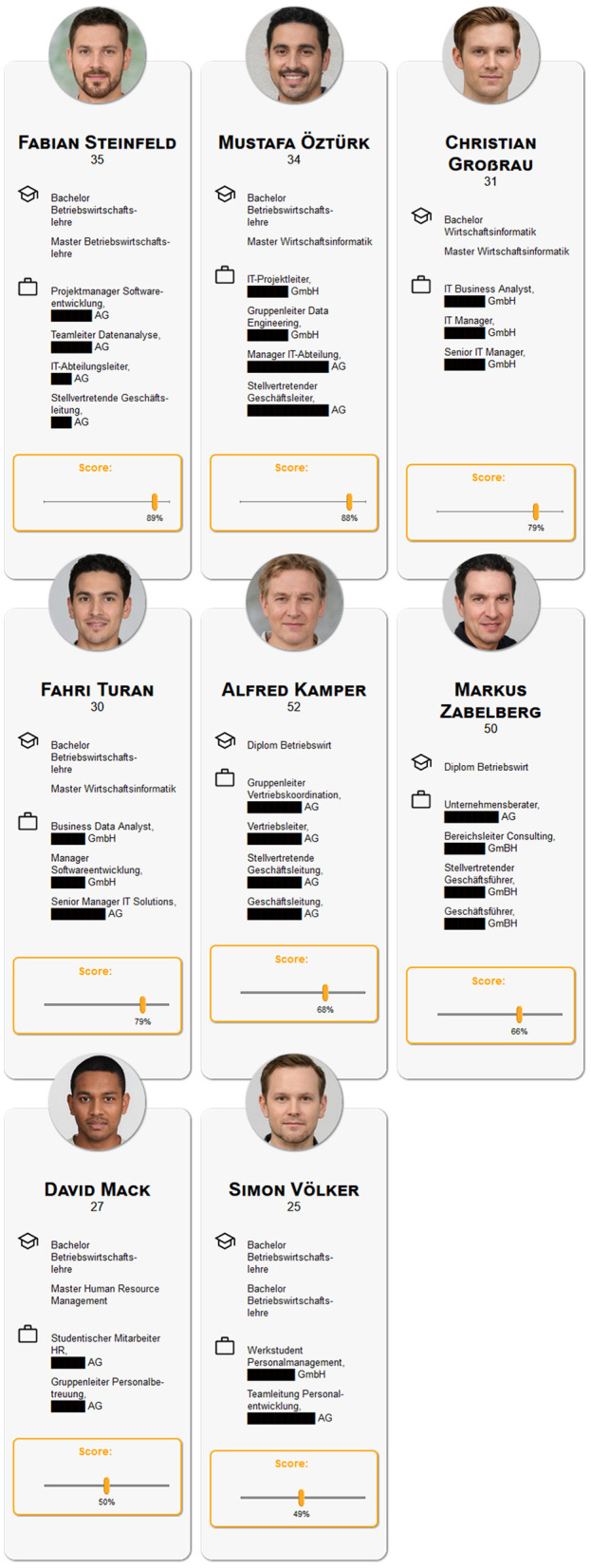
Screenshot of the presented applicants with their preset algorithmic scores in the non-biased condition. Photo by Generated Photos (https://generated.photos/).

**Figure 3:**
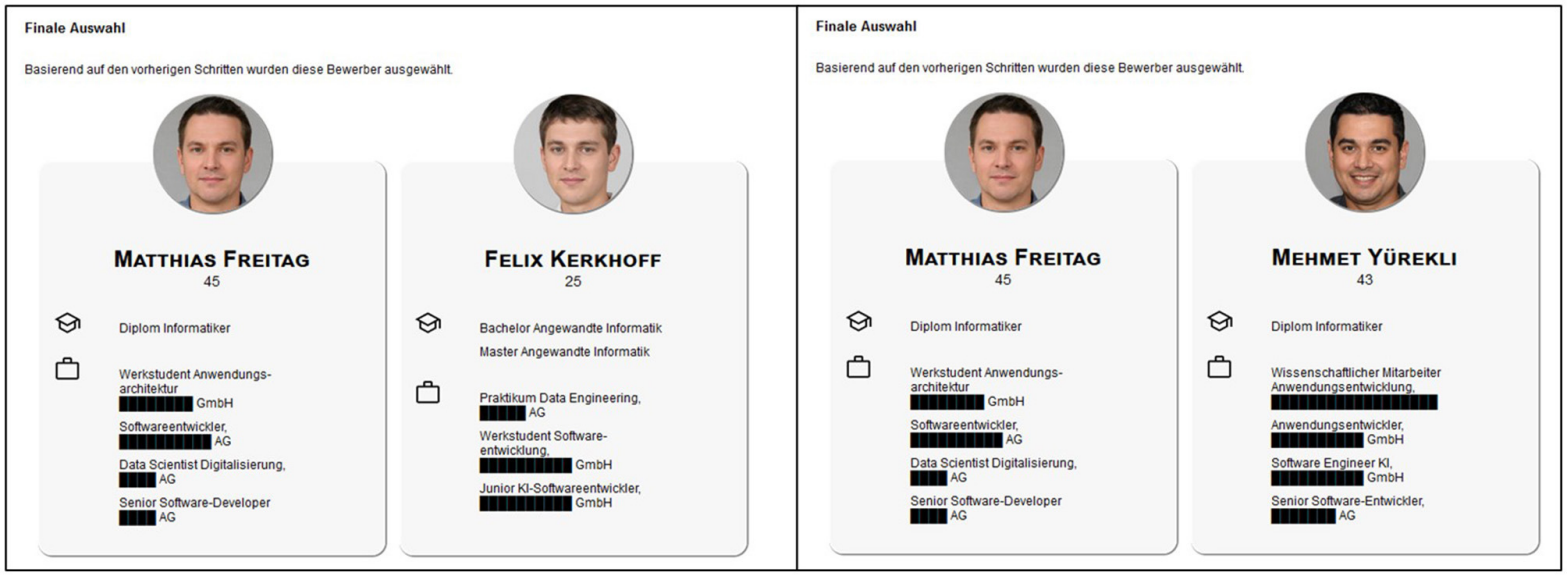
Final Selection of the job profile “Software Developer,” on the left side the biased version on the right side the non-biased version. Photo by Generated Photos (https://generated.photos/).

Participants were invited to test a hiring algorithm that screened applicants for two open positions: A manager position (Manager AI/Machine Learning) and a software developer position (Software Developer Digitalization and AI). Participants read a job description (for full translated descriptions see OSF project: DOI 10.17605/OSF.IO/KW8G9) and responded to an attention check asking participants for the title of the job position. Following, participants were reminded again with a short description of the job requirements (e.g. for Manager: “University degree in business or IT or relevant training with relevant professional experience; several years of management experience in the IT environment, ideally in dealing with artificial intelligence architectures and/or data engineering”) and were presented eight short descriptions of applicants and their qualifications, their photo, name, and age, and a numerical score generated by the simulated algorithm indicating the computed fit for the job (0–100%) on a slider scale (cf. Figure 1 for the biased version and Figure 2 for the not biased version of job profile Manager). Participants could adjust these scores as they saw fit. After completing this step, participants remained on a holding page for 3 s, informing them that the final selection was being calculated. On the next page, a final selection of two applicants was presented (cf. Figure 3). The selection of applicants was predetermined and not, as the participants were led to believe, influenced by their adjustments of the scores. On this page, participants could either indicate that they were satisfied with the selection or indicate that they would have preferred to select different applicants by choosing one of the displayed options that they would like to weigh more in the computation (e.g., younger applicants). This process was repeated for the respective other job. Presentation of the two positions and the respective interaction was randomized and in the following the two hiring rounds are referred to as *manager* round or *software developer* round. The job descriptions were compiled from genuine job advertisements.

To ensure that all participants are able to judge the applicants without the requirement of specific domain knowledge information about the candidates was very limited. As stated above information included photo, name, and age as well as university degrees and previous positions. The university degrees were all of equal quality meaning all applicants for the “Manager AI” position had a bachelor's and master's degree in business administration, business Informatics or human-resource management or a combination thereof. The only distinguishing information was the number of previous positions in IT and their position within a company's hierarchy ranging from student assistant, group/team leader, head of IT/sales, vice CEO, to CEO. Hence, applicants with previous positions in IT were rated better than those with positions outside of IT (e.g., sales), applicants with experience in higher positions were rated higher (e.g., CEO/Vice-CEO > group leader). The difference between the two applicants within one matched pair was often as minor as variations in German grammar or the use of the German or English term for the same position (e.g., deputy management vs. deputy manager; “Leiter” vs. “Manager”). A full translation of all applicant profile information such as study programs and positions can be found in the OSF project (DOI 10.17605/OSF.IO/KW8G9).

In each round eight applicants were introduced, of which five were presented as Germans without an immigrant background, two implied to have a Turkish background, and one was presented as having neither a German nor a Turkish background, using names and photos to convey ethnic origin. The names for all applicants were sourced from LinkedIn to ensure the use of realistic-sounding names, however, the actual profiles have no resemblance to any existing persons. The images used were AI-generated and taken from the free academic data set of generated photos (https://generated.photos/datasets/academic) and their conformity with the ethnicity they are meant to convey was pre-tested. Further, to avoid the effects of intersectional discrimination, particularly due to the stereotypically male jobs (Willemsen, 2002; Cheryan et al., 2015) only male applicants were included. Participants were informed that this was intentional due to testing of the algorithm under certain restrictions. The applicant profiles were created such that there are *matched pairs*, i.e., for each applicant, there exists an equally qualified second applicant. In the condition without algorithmic bias, the equally qualified applicants receive a nearly identical score (max. +/– 2% of the score assigned to one applicant in matched pair), while in the condition with algorithmic bias, the Turkish applicants receive a lower (–10%) score than their German counterparts with equal qualifications. In both hiring rounds, the first two matched pairs were composed of a German and a Turkish applicant, the third matched pair consisted of two German applicants, and the last matched pair of a German applicant and a Distractor (neither German nor Turkish background).

### 3.3 Measures

#### 3.3.1 Behavior during interaction—Reliance on algorithmic advice

Reliance is measured by assessing editing behavior concerning (1) the changes made to the scores assigned by the algorithm on sliders (score correction) and (2) the (dis-)satisfaction with the final selection of applicants. For the former, for each applicant in both rounds the *relative score corrections* participants made to the initial ranking was calculated by taking participant's scores and subtracting the initial algorithmic score. Positive values indicate a correction in favor of the candidate (correcting to a higher score), and negative values indicate a correction in disfavor of the candidate (correction to a lower score). To calculate the *magnitude of changes*, in a second step, the score corrections were transformed into absolute values and were summed up to enable a calculation of the magnitude of score corrections participants performed over all candidates regardless of whether these changes were positive or negative. The latter was operationalized by differentiating between participants who did not select that they were satisfied with the final selection and those who selected that they were satisfied with the final selection. This was determined using the item “I am satisfied with the selection.” When not satisfied with the final selection, the other nine possible options (radio button; forced choice) could be utilized to answer the item “I would like to weight the following factor more.” The nine were related to age, qualification, and diversity of applicants. For analysis of satisfaction with the final selection, answers were transformed into a new variable in which all nine alternative answering options were treated as “not satisfied,” resulting in two dichotomous variables for hiring round 1 and 2.

#### 3.3.2 Attitude toward and perceptions of algorithms

Participants' attitudes toward ADMs were assessed after the interaction with the algorithm using the German version of the *Attitudes toward Algorithm Scale* (ATAS) (Bock and Rosenthal-von der Pütten, 2023). The scale consists of 17 items in total rated on a 7-point-Likert scale from “do not agree at all” to “fully agree.” Items are grouped in three subscales of which we used two, namely *Objectivity* (six items, e.g., “Algorithms do not favor anyone,” Cronbach's α = 0.783), and *Ethicality* [seven items (all reverse-coded), e.g., “Algorithms should not make morally difficult decisions”, Cronbach's α = 0.615]. The sub-scale “Performance” which addresses that algorithms can analyze more data faster than humans can should not be affected by the current manipulation and thus was not used for reasons of test economy.

Based on the Fairness Dimensions introduced by Colquitt and Rodell (2015); Görlitz et al. (2023) developed a 16-item questionnaire on *Perceived Fairness of Algorithms* covering *procedural fairness, distributive fairness, interpersonal fairness*, and informational fairness. Participants answered these items before and after the interaction on a percentile slider from 0%: “Do not agree at all” to 100%: “Fully agree” and had the option not to answer by clicking “does not apply” (Cronbach's α = 0.857).

Participants' perception of the specific algorithm used in this study was measured. Participants rated the *Hiring Algorithm's Objectivity* on two items (“The algorithm suggested appropriate applicants” and “The algorithm has selected the objectively best applicants”; Cronbach's α = 0.832), the *Hiring Algorithm's Fairness* on one item (“The algorithm has evaluated all applicants fairly.”) and the *Hiring Algorithm's Performance* on seven items (“The algorithm... ...helped me to select suitable applicants faster; ...stopped working unexpectedly; ...had a reasonable response time; ...is easy to understand and use; ...has generated accurate results; ...has used sufficient parameters to make the decisions; ...has achieved the goal of supporting decision making in the selection of appropriate applicants”; Cronbach's α = 0.775) using a 5-point Likert scale ranging from “strongly disagree” to “strongly agree.” This *ad-hoc* generated items were partially adapted from a scale by Shafinah et al. (2010).

Finally, the experiment incorporated a query concerning the *Noticing of Algorithmic Bias* using two items asking participants to indicate whether the decisions made by the algorithm were biased against a certain subgroup (“Were the algorithm decisions biased in favor of Turkish applicants?”; “Were the algorithm decisions biased in favor of German applicants?”).

#### 3.3.3 Attitude toward Turkish People

Attitudes toward immigrants at a more general level are assessed by using two subscales of the German version of the Blatant and Subtle Prejudice Scale (Pettigrew and Meertens, 1995), measured on a 5-point Likert Scale ranging from “strongly disagree” to “strongly agree” for the Traditional Values Factor Items Subtle Scale (sample item: “Turkish People should not push themselves where they are not wanted”) and on a 4-point Likert scale ranging from “very often” to “never” for the Positive Emotion Factor Items Subtle Scale (sample item: “How often have you felt sympathy for Turkish people living here?”). In the following, the Traditional Values Factor Items Subtle Scale is referred to as *Emotions Negative* and the Positive Emotion Factor Items Subtle Scale as *Emotions Positive*. Moreover, we used a specific measure of stereotype endorsement designed specifically to assess individual stereotypes of Turkish males in managerial positions using the Turkish as Managers Scale (TAMS) by Baltes and Rudolph (2010). Statements such as “It is just as important for Turkish men as for German men that their work is interesting” are assessed on a 7-point Likert scale ranging from “strongly disagree” to “strongly agree”.

#### 3.3.4 Experience in human resources and machine learning

*Experience in Machine Learning* was assessed using three items (e.g., “I have used machine learning algorithms many times”) rated on a 7-point Likert scale ranging from “completely disagree” to “completely agree” (Cronbach's α = 0.907) and *Experience in Recruitment and Relevant Sectors* (information technology, software development) was assessed with the answering options (yes/no).

### 3.4 Participants

The study was approved by the ethics committee of the German Psychological Society. Participants were recruited by convenience sampling of German speakers (*n* = 132) and via Prolific (prolific.com) only targeting German speakers (*n* = 150). Participants' data were removed if data quality was low (*n* = 3) if either of the attention checks failed twice (*n* = 8) or if participants needed very little time to complete the questionnaire (<6 min, *n* = 3). At the end of the questionnaire participants were asked “In your honest opinion, should we use your data for our evaluation?”. One participant answered no and was excluded from the data analysis. Finally, seven participants were excluded because they had spent the most time of their lives outside of Germany. Since these participants were raised in a different country, potentially with different stereotypes, they were also excluded. The final sample consisted of 260 participants, with nine raised in Switzerland and currently living in Switzerland (*n* = 7) or Germany (*n* = 2), 23 having their origin and current residence in Austria, and 229 raised in Germany and current residence in Germany (*n* = 225), Austria (*n* = 3) or Portugal (*n* = 1). 147 participants self-identified as female, 108 as male, and five participants identified as diverse. Age ranged from 19 to 71 years (*M* = 30.57, *SD* = 10.03). The majority of participants were students (46%), employees (40%) or self-employed (6.5%). Other participants indicated to be in apprenticeship (*n* = 3), retired (*n* = 3), seeking work (*n*= 4), stay-at-home spouse (*n* = 5). Sixty-one percent hold a university degree, 8% a degree of vocational training, and 27 a high-school degree.

Previous experience of participants with topics related to this study was generally low: 78.5% reported having no experience in human resource management, 61% indicated not to have been involved in hiring processes, but 43% have acquaintances or family that are involved in recruitment. Most participants also had no experience with information technology (75.8%) or software development (88.5%). Prior Experience with machine learning algorithms was low (seven-point scale, *M* = 2.68, *SD* = 1.58 ).

An *a-priori* power analysis was conducted using G*Power (Faul et al., 2007) to determine the minimum sample size required for testing H1. Results indicated the required sample size to achieve 80% power for detecting a medium effect, at a significance criterion of α = 0.05, was *N* = 128 for a two-factor ANCOVA (Factor: bis/no-bias; Factor: Hiring round 1/2) with one covariate. Thus, the obtained sample size of *N* = 260 is adequate to test hypothesis 1.

## 4 Results

The statistical analyses in this study were computed using the statistics software SPSS Statistics 29. A significance level of 0.05 was used for all hypotheses.

### 4.1 Magnitude of change and direction of score corrections

Addressing ***H1a*** stating that individuals rely less on the algorithmic advice in the bias condition, an ANCOVA was calculated with condition (bias, no-bias) and hiring round (manager, software developer) as independent variables and magnitude of score corrections as dependent variable. After controlling for participant's attitudes toward Turkish people, results show a main effect of condition on magnitude of change, *F*_(1, 56)_ = 8.133, *p* = 0.005, partial η^2^ = 0.016, indicating that participants made more changes overall in the bias condition than in the no bias condition (cf. Table 1 for mean values and standard deviations). Further, a main effect of the hiring round was found, indicating that participants made more changes to the applicants' scores in the manager round compared to the software developer round, *F*_(1, 56)_ = 5.533, *p* = 0.019, partial η^2^ = 0.011. There was no interaction effect.

**Table 1:** Magnitude of changes to initial algorithmic scores across conditions and hiring rounds.

	**Manager**		**Software Dev**.		**Total**	
	***M***	***SD***	***M***	***SD***	***M***	***SD***
No bias	10.53	12.98	8.37	11.74	9.45	12.40
Bias	13.35	14.43	10.,28	12.09	11.82	13.37
Total	11.94	13.77	9.33	11.93	10.63	12.94

Separate ANCOVAS were run on the basis of the respective eight applicants with the condition and hiring rounds as independent variables and relative score corrections (positive/negative corrections) as dependent variables. After controlling for participants' attitudes toward Turkish people, results indicated the main effects of the condition for the first three applicants (one German, two Turkish, three German) and applicants 6 (German), and 7 (Distractor), indicating that participants made more changes in the bias than in the no-bias condition for these applicants. Moreover, there were main effects regarding the hiring round for six of the eight applicants, indicating that participants generally made more changes in the manager round than in the software developer round (cf. Table 2). No interaction effects emerged.

**Table 2:** Score corrections to all applicants across conditions and hiring rounds.

		**Manager**		**Software Dev**.		**Main effect condition**			**Main effect job**			**Interaction effect**		
		***M***	***SD***	***M***	***SD***	***F***	***p***	***eta***	***F***	***p***	***eta***	***F***	***p***	***eta***
*Applicant 1*	No bias	–3.71	10.50	–3.88	10.00	4.672	**.031**	.009	.068	.794	.000	.186	.667	.000
German	Bias	–6.42	14.99	–5.69	11.51									
*Applicant 2*	No bias	0.79	5.57	–2.00	7.82	18.073	**<.001**	.034	20.423	**<.001**	.039	.553	.457	.001
Turkish	Bias	4.72	11.32	0.83	8.53									
*Applicant 3*	No bias	–1.24	10.10	2.12	9.30	5.286	**.022**	.010	11.489	**.001**	.022	.020	.888	.000
German	Bias	–3.43	13.25	–0.34	10.44									
*Applicant 4*	No bias	–0.71	10.67	1.80	12.53	0.324	.570	.001	2.485	.116	.005	.738	.391	.001
Turkish	Bias	0.65	11.76	1.39	12.15									
*Applicant 5*	No bias	–8.63	16.62	–2.16	14.99	3.176	.075	.006	27.215	**<.001**	.050	.613	.434	.001
German	Bias	–11.61	19.49	–2.85	15.46									
*Applicant 6*	No bias	–7.74	15.92	–0.51	15.48	6.724	**.010**	.013	44.363	**<.001**	.080	3.416	.065	.007
German	Bias	–13.98	20.66	–1.19	16.07									
*Applicant 7*	No bias	–8.16	15.26	0.64	15.47	4.203	**.041**	.008	41.558	**<.001**	.075	.004	.947	.000
Distractor	Bias	–10.51	17.46	–1.52	14.90									
*Applicant 8*	No bias	–9.04	14.07	1.92	14.30	1.541	.215	.003	72.880	**<.001**	.124	.102	.749	.000
Distractor	Bias	–10.64	16.54	1.18	15,85	

Bold values mean significant effects under alpha level of 5 %.

Inspecting the descriptive statistics for the full sample results indicated that overall participants lowered algorithmic scores for German participants, while scores for Turkish applicants were raised. However, the range of applied changes was big from small adjustments to extreme changes. Moreover, the changes go in both directions, meaning that participants made positive as well as negative corrections for all applicants (cf. Table 3).

**Table 3:** Descriptive statistics for the score corrections for all applicants.

	***Ran***	***Min***	***Max***	***M***	***SD***	***VAR***
1. German	100	–89	11	–4.93	11.93	142.36
2. Turkish	100	–80	20	1.09	8.86	78.56
3. German	102	–79	23	–0.72	11.03	121.57
4. Turkish	95	–71	24	0.78	11.80	139.33
5. German	113	–68	45	–6.31	17.15	293.99
6. German	112	–66	46	–5.85	17.97	322.93
7. Distrac.	106	–50	56	–4.89	16.41	269.29
8. Distrac.	105	–49	56	–4.14	16.23	263.40

Finally, testing for selective adherence in following algorithmic advice (***H4***), correlation analyses were run between TAMS, Negative and Positive Emotions, and the two magnitude scores (manager and software developer). Only between the magnitude of score corrections in the manager round and Positive Emotions (*r* = –0.156, *p* = 0.012) a significant negative correlation was found. Individuals with more positive emotions toward the decision subjects exhibit less reliance. No effect emerged for negative emotions or prejudice against Turkish managers.

### 4.2 Satisfaction with final selection

Testing whether individuals rely less on the algorithmic advice in the bias condition (***H1b***), the influence of condition and of hiring round on participants' statements whether they are satisfied or dissatisfied with the final selection of applicants were analyzed using Chi-square tests. A significant influence of condition on satisfaction with the final selection was found, indicating that participants were more often dissatisfied in the bias condition [χ^2^ (1, *n* = 520) = 5.253, *p* = 0.022, Cramer's *V* = 0.10; cf. Table 4 for frequencies]. The hiring round, however, had no effect on satisfaction with the final selection (cf. Table 5 for frequencies). When dissatisfied with the final selection, participants could then choose one out of nine options as a factor that “I would like to weight the following factor more” indicating the most relevant reason for their dissatisfaction. These factors were related to age, qualification, or diversity (cf. Table 6 for all options). A noteworthy difference is that for the software developer, the factor of age was more important: More people wished to include younger applicants or applicants of more diverse ages. For the manager participants wished to include more applicants with more previous experience. Diversity was chosen to an equal amount for both profiles but was a less important factor than qualification.

**Table 4:** Frequencies for (dis-)satisfaction with final selection across conditions.

	**Condition**		
	**No bias**	**Bias**	**Total**
Satisfied	130	104	234
Not satisfied	130	156	286
Total	260	260	520

**Table 5:** Frequencies for (dis-)satisfaction with final selection across hiring round.

	**Hiring round**		
	**Manager**	**Software Dev**.	**Total**
Satisfied	127	107	234
Not satisfied	133	153	286
Total	260	260	520

**Table 6:** Frequencies of choices for factors that should be weighted more in final selection.

**Reason**		**Manager**	**Software developer**
Age	More younger applicants	3	38
	More older applicants	7	3
	More applicants of diverse age	6	12
Qualification	More applicants with high educational qualifications	7	7
	More applicants with alternative educational paths	10	15
	More young professionals	3	6
	More applicants with a lot of previous experience	62	39
Diversity	More applicants from different backgrounds	24	20
	More applicants with different previous experiences	11	13
Total		133	153

### 4.3 Noticing of the bias

Since the manipulation distinguishing the two conditions is based on the presence of an algorithmic bias (bias, *n* = 130) or the absence of such a bias (no-bias, *n* = 130), it was examined whether participants indicated to have observed such a bias in favor of German applicants in the two conditions (***RQ1***) and whether there are differences between conditions (***RQ2***). Using Chi-square tests the influence of condition on participants' statements to have noticed a bias (yes, no) regarding German applicants and Turkish applicants was analyzed. A significant influence of condition on noticing bias in favor of German applicants was found [χ^2^ (1, *n* = 260) = 30.007, *p* < 0.001, Cramer's *V* = 0.34; cf. Table 7 for frequencies] showing that participants in the bias condition reported more often than in the no-bias condition to have observed a bias in favor of German applicants. As a cross-check, we also looked into the distractor item that asked whether participants noticed a bias in favor of Turkish applicants (cf. Table 8). No such difference between conditions emerged for noticing a bias in favor of Turkish applicants, which was indeed only mentioned by 15 participants, while 69 participants reported to have noticed a bias in favor of German participants. Remarkably, in the bias condition, only 41% (54 participants) reported to have noticed a bias.

**Table 7:** Frequencies of reported noticing of bias in favor for German applicants.

	**Noticed bias**		
	**Yes**	**No**	**Total**
No bias	15	115	130
Bias	54	76	130
Total	69	191	260

**Table 8:** Frequencies of reported noticing of bias in favor of Turkish applicants.

	**Noticed bias**		
	**Yes**	**No**	**Total**
No bias	7	123	130
Bias	8	122	130
Total	15	245	260

Next, it was explored whether there is a connection between individual's noticing of the algorithmic bias and their reliance on the algorithm (***RQ3***). For those participants who were in the bias condition and thus experienced a bias (*n* = 130), it was tested whether individuals relying less on the algorithm (showing more editing behavior) are more likely to notice the bias (***H2a***) with a regression utilizing Magnitude of Change as a predictor. Assumptions, including linearity of the logit, were tested and a binary logistic regression was calculated. Results indicate that participants in the bias condition are not more likely to indicate a bias when they have made more changes in the manager [χ^2^(1) = 0.41, *p* = 0.524] or software developer hiring round [χ^2^(1) = 0.37, *p* = 0.542]. It was also tested whether participants who reported being dissatisfied with the final selection (satisfied, dissatisfied) of each hiring round were more likely to have noticed the bias (yes, no) addressing ***H2b*** (only based on participants in the bias condition, *n* = 130). Using Chi-square tests a significant effect was found, suggesting that (for both hiring rounds) participants who were dissatisfied with the final selection reported more often to have noticed a bias than could be expected by the data [manager: χ^2^ (1, *n* = 130) = 9.969, *p* = 0.002, Cramer's *V* = 0.28; Software Developer: χ^2^ (1, *n* = 130) = 12.05, *p* < 0.001, Cramer's *V* = 0.304; cf. Tables 9, 10 for frequencies]. Noteworthy is that the effect is smaller for dissatisfaction with the manager selection.

**Table 9:** Frequencies of reported noticing of bias dependent of (dis)satisfaction with final selection in hiring round 2 (manager); based on participants in biased condition (*n* = 130).

		**Noticed bias**		
		**Yes**	**No**	**Total**
*Satisfied*	Observed frequencies	11	36	47
	Expected frequencies	19.5	27.5	47
*Dissatisfied*	Observed frequencies	43	40	83
	Expected frequencies	34.5	48.5	83
*Total*	Observed frequencies	54	76	130
	Expected frequencies	54	76	130

**Table 10:** Frequencies of reported noticing of bias dependent of (dis)satisfaction with final selection in hiring round 1 (software developer); based on participants in biased condition (*n* = 130).

		**Noticed bias**		
		**Yes**	**No**	**Total**
*Satisfied*	Observed frequencies	14	43	57
	Expected frequencies	23.7	33.3	57
*Dissatisfied*	Observed frequencies	40	33	73
	Expected frequencies	30.3	42.7	73
*Total*	Observed frequencies	54	76	130
	Expected frequencies	54	76	130

With point-biserial correlations we tested whether participants with more positive attitudes (i.e., continuous variables TAMS, Emotions Positive, Emotions Negative) are more likely to notice the bias (coded: 1 = bias noticed; 2 = bias not noticed) in favor for German applicants than participants with a more negative attitude ***H5***, but found results show no significant correlation between Emotions Positive (*rpb* = –0.037, *p* = –0.549) or TAMS (*rpb* = –0.064, *p* = 0.306) with noticing the bias. Only Emotions Negative was significantly related to the noticing of the bias (*rpb* = 0.183, *p* = 0.003) suggesting that higher negative emotions are related to not noticing the bias more often. Following the correlation analysis, a logistic regression using entry method was performed to ascertain the effects of participants' attitudes (negative Emotions, positive Emotions, TAMS) on the likelihood that they notice the bias. The logistic regression model was not statistically significant when including all three variables [χ^2^ (8, 260) = 14.299, *p* = 0.074]. Examining the included variables only the scale negative Emotions was a significant predictor (cf. Table 11). Following, a logistic regression with the forward stepwise method was performed resulting in a statistically significant model including negative Emotions, χ^2^ (8, 260) = 9.067, *p* = 0.003. The model explained 5.0% (Nagelkerke *R*^2^) of the variance in noticing the bias and correctly classified 73.5% of cases.

**Table 11:** Summary of logistic regression analysis predicting noticing the bias.

		**95% CI for odds ratio**		
	*B* **(SE)**	**Lower**	**Odds ratio**	**Upper**
Constant	0.939 (2.15)			
Negative emotions	0.691* (0.22)	1.29	2.00	3.09
Positive emotions	–0.470 (0.26)	0.38	0.63	1.04
TAMS	–0.038 (0.27)	0.57	0.96	1.64

### 4.4 Attitude toward and perceptions of algorithms

ANOVAS with the condition as the independent variable and the ATAS subscales Objectivity and Ethicality, as well as the scale Fairness of Algorithms as dependent variables, yielded no significant effects. General attitudes toward algorithms were not affected by experiencing a biased algorithm (cf. Table 12). It was then explored whether the interaction with the biased algorithm had an influence on participants' evaluations of this specific hiring algorithm that they interacted with. Indeed, ANOVAS with the condition as independent and Objectivity, Fairness, and Performance as dependent variable showed that participants rated the algorithm more fair, more objective, and better in performance in the no-bias condition compared to the biased condition (cf. Table 13). Addressing the hypothesis that individuals who noticed a bias are more likely to rate algorithms more negatively in general (***H3a***) and especially regarding fairness (***H3b***) ANOVAS were conducted, this time however, only considering participants in the bias condition. Noticing the bias [noticed yes (*n* = 54); noticed no (*n* = 76)] was used as independent variable and the general attitudes and evaluation of the specific algorithm were used as dependent variables. Significant effects for all dependent variables except for Ethicality were found, meaning that participants who noticed the bias had more negative attitudes toward algorithms in general (cf. Table 14) and evaluated the algorithm that they had experienced as being less objective, less fair and worse in performance (cf. Table 15).

**Table 12:** Influence of condition on general attitudes toward algorithms.

		*M*	**SD**	*F*	*p*	**eta**
Objectivity	No bias	4.67	1.44	2.183	.141	.008
(ATAS)	bias	4.41	1.41			
Ethicality	No bias	5.06	1.19	0.127	.722	.000
(ATAS)	bias	5.11	1.06			
Fairness	No bias	46.47	14.88	0.085	.771	.000
Of Algo.	bias	45.93	14.78			

**Table 13:** Influence of condition on evaluation of the specific hiring algorithm.

		*M*	*SD*	*F*	*p*	**eta**
Objectivity	No bias	3.97	0.87	20.386	.001	.073
	Bias	3.43	1.06			
Fairness	No bias	3.67	0.98	10.360	.001	.039
	Bias	3.24	1.17			
Performance	No bias	4.03	0.59	10.427	.001	.039
	Bias	3.79	0.63			

**Table 14:** Influence of noticing the bias on general attitudes toward algorithms.

		***M***	***SD***	***F***	***p***	***eta***
Objectivity	Yes	3.74	1.32	24.348	<.001	.160
(ATAS)	No	4.89	1.29			
Ethicality	Yes	5.05	1.00	0.237	.627	.002
(ATAS)	No	5.15	1.11			
Fairness	Yes	38.72	13.70	26.297	<.001	.170
of Algo.	No	51.05	13.37			

**Table 15:** Influence of noticing the bias on evaluation of the specific hiring algorithm.

		***M***	***SD***	***F***	***p***	***eta***
Objectivity	Yes	2.73	1.05	57.249	<.001	.309
	No	3.92	0.74			
Fairness	Yes	2.44	1.06	63.508	<.001	.332
	No	3.80	0.88			
Performance	Yes	3.39	0.57	49.698	<.001	.280
	No	4.07	0.51			

## 5 Discussion

The research undertaken looks into the interplay between human and algorithmic biases. Specifically, whether such algorithmic biases are noticed, and whether the attitude of individuals toward algorithms and toward the group negatively affected by the bias has an influence on this noticing. Further, it was investigated whether the reliance of individuals on such a biased algorithm is reduced, and if this, in turn, is related to the attitude toward the affected group of individuals. The majority of the hypotheses put forward in this paper could be supported. Below, we discuss the findings in relation to existing literature and their implications for decision-making in the age of automation.

### 5.1 The presence of algorithmic bias affects behavior during interaction

As put forward by the first hypothesis, results indicate that individuals in the condition with an algorithmic bias indeed made more changes to the algorithm's initial ratings of applicants (***H1a***), and were less satisfied with the final selection (***H1b***), which is in line with previous automation bias literature concerning users' behavior upon encountering erroneous algorithms (Dietvorst et al., 2015; Yu et al., 2017). Additionally, scores were adjusted significantly more in the manager round than in the software developer round, while there was no difference in satisfaction with the final selection. This difference in reliance depending on the job (manager vs. software developer) could be related to varying levels of uncertainty: In situations of judgmental uncertainty, people often resort to simplifying heuristics (Tversky and Kahneman, 1974). The anchoring effect is apparent in the fact that numeric estimates are assimilated to a previously considered standard, the anchor (Enough and Mussweiler, 2001). It has been suggested that the size of this anchoring effect increases with uncertainty (Jacowitz and Kahneman, 1995). Participants likely knew the terms used in relation to the manager position and the associated responsibilities due to it being a long-established profession with which most people are likely to be familiar. The opposite may be true for the software developer position, which might lead to increased uncertainty about the candidates' qualifications, fostering the reliance on heuristics. As a result, participants might have made fewer changes to the scores due to higher reliance on the provided algorithm's rating, the anchor. Following the argumentation of Shaikh and Cruz (2023) human responses toward algorithmic decision-support systems may thus also be dependent on contextual factors such that the human choice to rely on these systems is a heuristic which emerges as an artifact of constraining circumstances. Regarding the research question asking if the presence of an algorithmic bias has an influence on individuals' reliance on the algorithm (***RQ2***), it can be summarized that individuals in our study seem to rely less on a biased algorithm than on a non-biased algorithm. As overall participants lowered algorithmic scores for German participants and raised scores for Turkish applicants, results also indicate that the changes made were *generally* countering the bias although that is not true for every individual participant.

### 5.2 Stereotypical attitudes affect adaptation of applicant scores

In terms of individuals' attitudes in this process, results indicate that individuals with more positive emotions toward the decision subjects exhibit less reliance and thus make more changes (***H4***). While several previous studies have found no relationship between explicit attitudes and discrimination in hiring (Stewart and Perlow, 2001; Krings and Olivares, 2007, cf.), this difference in results might be due to the provision of an already existing assessment. In the context of the Elaboration-Likelihood Model, an algorithm score can represent a peripheral cue that might override qualified information such as applicant qualifications when peripheral processing occurs (Forret and Turban, 1996). When people have the opportunity and motivation to assess consequences, they reflect upon their conscious attitudes relevant to the decision, whereby explicit attitudes primarily influence responses (Dovidio and Gaertner, 2000). In the current study, explicit attitude might have thus had an effect on the mitigation of scores because people were considering their conscious attitude, and not relying on e.g., the anchor provided by means of the algorithm's rating. Further, Gattino et al. (2008) suggest that people could use algorithms to legitimize their discrimination, insofar as individuals who do not discriminate the outgroup openly may do so if there is a socially acceptable way of doing so, e.g., by following algorithm advice. Thus, while in the mentioned studies participants had to do the evaluation independently and could not use an already assigned score to justify their choices, in this study, the anchor was already biased enabling individuals with a more negative attitude to essentially passively discriminate against Turkish applicants.

### 5.3 Noticing algorithmic biases and adaptation of applicant scores

Concerning the question of whether algorithmic biases are noticed (***RQ1***), first, it can be noted that while more participants in the bias condition reported noticing a bias in favor of German applicants than participants in the no-bias condition, only 41% of participants in the bias condition correctly identified the algorithm disadvantaging Turkish applicants. Thus, the fraction of participants who did successfully identify the bias in this study is lower than would be desirable. Especially, when considering that humans are envisioned to take the role of a human-in-the-loop which means to review the outcome of algorithmic decision-making and to intervene if necessary, such low detection rates would be problematic because they further perpetuate the problem organizations want to solve with using algorithms (Fejerskov, 2021; Mahmud et al., 2022). The goal of more objective and bias-free hiring decisions would not be met. There are many factors that could have an influence on the (lack of) noticing of such a bias. For one, the participants in this study had no information about how the scores for the applicants were calculated which makes the algorithm presented to them very opaque. In this respect, Janssen et al. (2022) note that people are particularly prone to not detecting biases if the algorithm used is non-transparent. However, it is also possible that an observation of the unequal treatment of German and Turkish applicants indeed occurred but was not labeled as a bias. This is conceivable because, for example, in studies of heteronormativity in schools, it has been observed that educators often deny its existence and affirm schools as neutral despite observing instances of its display, thereby effectively rendering it invisible (Atkinson and DePalma, 2009). Thus, our results support the view that the supposed objectivity of algorithms could lead to individuals missing instances of biases or refraining from labeling such observed instances as biased, as it may not fit with algorithms' promise of neutrality. This highlights the need for more transparency in algorithmic-decision making in hiring also because this type of system is considered a “High Risk AI System” according to article 6 paragraph 2 in the EU regulation on harmonized rules on artificial intelligence (EU AI Act) and “High-risk AI systems shall be designed and developed in such a way as to ensure that their operation is sufficiently transparent to enable deployers to interpret a system's output and use it appropriately.” according to article 13 paragraph 1. Providing future users with a scenario like we used it in our study might be suitable to raise awareness for biases in ADMs and the need to be attentive. In a similar study on biased decision-making in hiring we interviewed participants after the interaction with the biased algorithm regarding their experience and found that engaging in such an activity made participants reflect (Görlitz et al., 2023). However, although transparency is called for, more research is needed to explore how effective different approaches to transparency are in informing decision makers in hiring and in preventing selective adherence.

Regarding the assumed connection between individual's noticing of the algorithmic bias and their reliance on the algorithm (***RQ3***)(***H2a***), we found that individuals in the bias condition are not more likely to indicate a bias when they have made more changes. This might further suggest that people might have noticed the bias but failed to acknowledge or label it as such. Such lack of recognition can lead to a failure to appropriately address such biases which may also be related to the supposed objectivity or neutrality of algorithms (Benjamin, 2019): People's beliefs about the unbiased nature of algorithms may hinder the detection or flagging of biases because people tend to ignore or avoid information that contradicts or refutes their beliefs (Koriat et al., 1980). It is also possible that participants did not recognize the biased predictions explicitly, but implicitly (Luong et al., 2021): Individuals may have noticed the discrepancy between scores and qualifications and even made the “appropriate” adjustments to match the scores to the actual qualifications but failed to recognize the bias against a particular group of people. In other words, they might have noticed the algorithm's erroneousness without noticing the systematic bias.

Addressing ***H2b*** it was found that participants who reported to be dissatisfied with the final selection are more likely to have noticed the bias. It is possible that an effect in terms of noticing of the bias could be found for the final selection but not for the adjustment of scores because participants had to provide feedback to the algorithm if they were not satisfied with the final selection. Accordingly, participants who adjusted the scores without one specific reason might be more likely to be satisfied with the final selection, as they can only virtually reject the algorithm's selection since there is no option to adjust it, i.e., to reduce their reliance. Thus it can be assumed that those who did had a compelling reason for their dissatisfaction that they were consciously aware of. These results may also imply that participants not relying on the algorithm's final selection were more motivated and engaged in more high-elaborative processing as such reasoning requires mindful and thorough processing of information (Forret and Turban, 1996). Regarding the research question of whether there is a connection between individual's noticing of the algorithmic bias and their reliance on the algorithm (***RQ3***) it can be summarized that the adjustment of the scores does not affect the noticing of the bias, while the indication of (dis-)satisfaction with the final selection does. The former may be the case as participants might have exhibited editing behavior by reducing the discrepancy of scores and qualifications without noticing the systematic bias. The effect of the latter may be due to participants engaging actively in giving feedback and thereby exerting higher-elaborative processing and paying more attention to the information, which could in turn lead to them being more likely to notice the bias.

### 5.4 Stereotypical attitudes affect noticing of algorithmic bias

Regarding the role of the attitude toward the decision subjects in noticing the bias (***H5***) results suggest that lower negative emotions toward the decision subjects are related to noticing the bias more often although this influence is limited. Thus regarding ***RQ5***, it can be summarized that a more negative attitude seems to be related to both increased reliance and reduced noticing of biases. Prior research has noted that in order to confront prejudice, it is critical that action is recognized as discriminatory (e.g. Ashburn-Nardo et al., 2008). For instance, it was demonstrated that men who endorsed feminist beliefs were more aware of sexism, and specifically, the more men recognized that society is biased toward supporting patriarchy, the more incidents of sexism they identified (Hyers, 2007). Similarly, those who have a more positive attitude toward immigrants and acknowledge discrimination against low-status groups may be more likely to notice occurrences of bias against them, while individuals less empathetic toward targeted individuals may be less likely or willing to notice discrimination (Basford et al., 2014; Davis and Gentlewarrior, 2015). Further, if such systems are expected to be objective and free of bias, individuals not concerned about issues of discrimination and with negative attitudes toward minority groups could potentially worsen this effect as automated decisional aids tend to create a “moral buffer” (Cummings, 2006, p. 10), resulting in a diminished sense of moral agency, personal responsibility, and accountability for the user, which may inhibit individuals not sensitive to discrimination further in the detection of biases.

### 5.5 Evaluation of (non)biased algorithms

Individuals who noticed a bias are more likely to rate algorithms more negatively in general (***H3a***) and especially regarding fairness (***H3b***). Participants who noticed the bias had more negative attitudes toward algorithms in general. Sartori and Bocca (2023) found that individuals with higher levels of competence hold more positive opinions about AI. As previous research has already demonstrated that information about potential system errors or shortcomings of an algorithm can reduce automation bias (Kupfer et al., 2023), more competent users may be less susceptible to automation bias. Since a reduction in automation bias due to information about system deficiencies also implies reduced reliance in cases of automation failure, this could suggest that such information makes people more likely to notice such failures and consequently reduce their reliance when they occur. While in this study participants were not instructed about the potential bias of the algorithm, Alon-Barkat and Busuioc (2023) suggest that enhanced awareness of discrimination and algorithmic biases may diminish the effects of selective adherence. The current results may thus indicate that people who notice such biases are generally more skeptical of algorithms and could therefore have a more accurate perception of their potential faults, which could in turn help them to spot such faults more easily. Further participants evaluated the algorithm that they had experienced as being less objective, less fair and worse in performance. This is in line with previous research indicating that an algorithm's outputs and process characteristics impact fairness perceptions associated with the algorithm (Lee et al., 2017).

In summary, this research contributes to the existing literature on automation bias by examining its occurrence in recruitment processes in relation to ethnic discrimination in a study with laypeople. Algorithmic hiring assessments have already been described as undermining applicants' ability to construct and negotiate the representations through which the employer views them (Aizenberg et al., 2023). Additionally, this study provides initial empirical support that this representation can be further impacted by biases of such systems. Admittedly, our study included laypeople and no professionals, but the task at hand was designed in such a matter that it did not require expert knowledge. Given that our participants were able to execute the task at hand in a reasonable manner, the results underline that humans might not be fully reliable in attending to their oversight tasks. Consequently, efforts to bring fairness to discriminatory AI solutions must be seen as a matter of justice, where the trust among actors is ensured through the technology design. Such systems should be auditable and evaluated by social groups to ensure that they do not perpetuate discrimination (Varona and Suarez, 2023). This may emphasize the importance of propositions that the mitigation of risks associated with AI is best addressed at the level of institutional design (Laux, 2023). Schuett (2023) for instance notes that companies using AI could implement the three lines of defends model to increase the effectiveness of risk management practices so that individual, collective, or societal harm can be prevented.

### 5.6 Limitations

We want to discuss some limitations of this study. The sample in our study consists of laypeople. Only about 40% of participants have been involved in hiring processes before. We designed the study with having this in mind, i.e. providing very limited and general information regarding the candidate profiles including only degrees and previous work experience in terms of years and position in previous companies. Hence, participants did not have to evaluate specific expertise or skill sets. However, it can well be that experienced recruiters might judge the AI recommendations differently because they might have received training to identify biases in decisions they shall supervise or to avoid having biases themselves in decision-making processes. Unfortunately, our literature review on human biases in hiring citing very recent work on sexism, racism, ageism, or discrimination against members of the LGBTQ+ community, disabled people or mentally ill people 2.1 suggests that biases are still prevalent in hiring processes. Additionally, many white papers funded and published by the German government further demonstrate a still existing prevalence for racism in hiring in Germany (e.g., Schneider, 2014; Koopmans et al., 2018). Hence, it is likely that similar effects as detected with our laypeople sample would also emerge with an expert sample. This assumption has to be tested in future studies of course highlighting the need to replicate our results with expert samples.

Regarding the noticing of the bias, it is not possible to exactly determine from the data obtained when the bias was noticed, i.e., in which round (manager, software developer) or in which part of each round (editing of scores; final selection), or just at the end of the questionnaire when being confronted with the question. Moreover, participants were explicitly asked whether they noticed a bias, therefore the results make it impossible to distinguish between those who actually noticed a bias and those who only reported having noticed one suggesting they did because of social desirability. Participants who did not notice a bias might have selected that they did due to feelings of guilt and the desire to compensate (Iyer et al., 2003). The present study represents a first attempt to examine the actual noticing of such algorithmic biases in the recruitment of job applicants, thus, further research addressing this issue could shed more light on this matter, for example by conducting an additional qualitative analysis in which participants are asked to describe what specifically they noticed.

A further limitation concerns the not hypothesized differences found between the two rounds indicating that the rounds are not fully comparable, which could affect the generalizability of the results. It should be further studied whether there are indeed differences based on jobs and whether uncertainty or other unconsidered factors played a role. We explained these differences with more uncertainty regarding the software developer position which might have caused a stronger anchoring effect, meaning that participants stayed closer to the anchor rating provided by the system. In future work this assumption can be tested by directly asking participants how easy the judgments were for them and how confident they are that they can judge adequately.

At the end of the study, we asked participants whether they noticed a bias. To keep the wording consistent for both target groups (Turkish minority and German majority) we asked: Were the algorithm's decisions biased in favor of German applicants? And: Were the algorithm's decisions biased in favor of Turkish applicants? In future studies, we would also include the linguistic counterparts (“biased against”) for both target groups since studies on valence framing suggest that negative framing seems to yield stronger results than its positive counterpart (e.g., Bizer and Petty, 2005). Finally, the scales to measure attitudes toward Turkish people, for instance, the Blatant and Subtle Prejudice Scale, are indeed not subtle in measuring attitudes. Future studies might consider to include scales that are less confrontational and thus maybe better suited to assess negative attitudes about minorities such as the syndrome of group-focused enmity scale (Zick et al., 2008).

## 6 Conclusion

When algorithms are thought to have the potential to eliminate human bias is it important to determine whether algorithmic biases are noticed and/or selective adherence is occurring, especially, as evidence of systematic algorithmic bias accumulates and human decision-makers in-the-loop are seen as critical checks (Alon-Barkat and Busuioc, 2023). Therefore, it is important to examine the extent to which human decision-makers can actually act as effective decision facilitators in this capacity and successfully insure against such risks posed by algorithmic decision-support systems. Our results suggest that biased systems impact human reliance in a similar way as systems erring unsystematically, meaning we find evidence for automation bias as well as for selective adherence. Explicit attitudes toward the decision subjects could partially predict the reliance in terms of editing of scores since individuals with a more positive explicit attitude appear to be more likely to notice the bias and mitigate it more. However, without explicitly noticing the bias, i.e. acknowledging the systematic bias, participants showed editing behavior as if reacting to an erroneous system. Although this study may in general support the notion of automation bias, the most important contribution may be that the conducted research raises a variety of intriguing questions for future study. The noticing of algorithmic biases warrants further research, particularly because the results of this study suggest that even relatively blatant biases can be overlooked. At the same time, an encouraging, tentative take-away emerges from the investigation: When individuals are more conscious and alert to such risks this may affect potential attenuation in noticing of algorithmic biases, which could be a promising path for mitigation of such problems.

## Data availability statement

The datasets presented in this study can be found in online repositories. The names of the repository/repositories and accession number(s) can be found at: https://osf.io/kw8g9/.

## Ethics statement

The studies involving humans were approved by Ethic Committee of the German Psychological Association. The studies were conducted in accordance with the local legislation and institutional requirements. The participants provided their written informed consent to participate in this study.

## Author contributions

AR: Conceptualization, Data curation, Formal analysis, Funding acquisition, Investigation, Methodology, Project administration, Supervision, Writing – original draft, Writing – review & editing. AS: Conceptualization, Investigation, Methodology, Writing – original draft, Writing – review & editing.

## References

[B1] Aizenberg E. Dennis M. J. van den Hoven J. (2023). Examining the assumptions of AI hiring assessments and their impact on job seekers' autonomy over self-representation. AI Soc. 1–9. 10.1007/s00146-023-01783-1 PMC11968453 40191164

[B2] Alberdi E. Strigini L. Povyakalo A. A. Ayton P. (2009). “Why are people's decisions sometimes worse with computer support?” in Computer Safety, Reliability, and Security, eds. B. Buth, G. Rabe, and T. Seyfarth (Berlin: Springer), 18–31. 10.1007/978-3-642-04468-7_3

[B3] Alelyani S. (2021). Detection and evaluation of machine learning bias. Appl. Sci. 11:6271. 10.3390/app11146271

[B4] Alon-Barkat S. Busuioc M. (2023). Human-AI interactions in public sector decision making: “automation bias” and “selective adherence” to algorithmic advice. J. Public Adm. Res. Theory 33, 153–169. 10.1093/jopart/muac007

[B5] Ashburn-Nardo L. Morris K. A. Goodwin S. A. (2008). The confronting prejudiced responses (CPR) model: applying cpr in organizations. Acad. Manag. Learn. Educ. 7, 332–342. 10.5465/amle.2008.34251671

[B6] Atkinson E. DePalma R. (2009). Un-believing the matrix: queering consensual heteronormativity. Gend. Educ. 21, 17–29. 10.1080/09540250802213149

[B7] Axt J. R. Ebersole C. R. Nosek B. A. (2014). The rules of implicit evaluation by race, religion, and age. Psychol. Sci. 25, 1804–1815. 10.1177/0956797614543801 25079218

[B8] Baekgaard M. Serritzlew S. (2016). Interpreting performance information: motivated reasoning or unbiased comprehension. Public Adm. Rev. 76, 73–82. 10.1111/puar.12406

[B9] Baltes B. B. Rudolph C. W. (2010). Examining the effect of negative Turkish stereotypes on evaluative workplace outcomes in Germany. J. Manag. Psychol. 25, 148–158. 10.1108/02683941011019357

[B10] Barocas S. Selbst A. D. (2016). Big data's disparate impact. SSRN Electron. J. 10.2139/ssrn.247789928632438

[B11] Baron R. A. (1991). Positive effects of conflict: a cognitive perspective. Empl. Responsib. Rights J. 4, 25–36. 10.1007/BF01390436

[B12] Basford T. E. Offermann L. R. Behrend T. S. (2014). Do you see what I see? Perceptions of gender microaggressions in the workplace. Psychol. Women Q. 38, 340–349. 10.1177/0361684313511420

[B13] Batinovic L. Howe M. Sinclair S. Carlsson R. (2023). Ageism in hiring: a systematic review and meta-analysis of age discrimination. Collabra Psychol. 9:82194. 10.1525/collabra.82194

[B14] Becker G. S. (1995). The economics of discrimination. Economics research studies of the Economics Research Center of the University of Chicago, 2nd Edn. Chicago, IL: University of Chicago Press.

[B15] Benjamin R. (2019). Assessing risk, automating racism. Science 366, 421–422. 10.1126/science.aaz3873 31649182

[B16] Bizer G. Y. Petty R. E. (2005). How we conceptualize our attitudes matters: the effects of valence framing on the resistance of political attitudes. Polit. Psychol. 26, 553–568. 10.1111/j.1467-9221.2005.00431.x

[B17] Bjørnshagen V. Ugreninov E. (2021). Disability disadvantage: experimental evidence of hiring discrimination against wheelchair users. Eur. Sociol. Rev. 37, 818–833. 10.1093/esr/jcab004

[B18] Blanco F. (2017). “Cognitive bias” in Encyclopedia of Animal Cognition and Behavior, eds. J. Vonk, and T. Shackelford (Cham: Springer International Publishing), 1–7. 10.1007/978-3-319-47829-6_1244-1

[B19] Bock N. Rosenthal-von der Pütten A. (2023). “Exploring the contextuality of attitudes towards algorithmic decision-making: Validation of the newly developed universal attitudes towards algorithms scale (ATAS),” in HMC Pre-Conference of the 73rd Annual International Communication Association (ICA) Conference (Toronto, ON).

[B20] Bogen M. Rieke A. (2018). Help wanted: An examination of hiring algorithms, equity, and bias. Washington, DC: Upturn.

[B21] Bornstein S. (2018). Antidiscriminatory algorithms. Ala. L. Rev. 70:519. Available online at: https://ssrn.com/abstract=3307893

[B22] Calice M. N. Bao L. Freiling I. Howell E. Xenos M. A. Yang S.. (2021). Polarized platforms? How partisanship shapes perceptions of “algorithmic news bias”. New Media Soc. 25, 2833–2854. 10.1177/14614448211034159

[B23] Carlsson M. Rooth D.-O. (2007). Evidence of ethnic discrimination in the Swedish labor market using experimental data. Labour Econ. 14, 716–729. 10.1016/j.labeco.2007.05.001

[B24] Carter E. R. Murphy M. C. (2015). Group-based differences in perceptions of racism: what counts, to whom, and why? Soc. Personal. Psychol. Compass 9, 269–280. 10.1111/spc3.12181

[B25] Chamorro-Premuzic T. Winsborough D. Sherman R. A. Hogan R. (2016). New talent signals: Shiny new objects or a brave new world? Ind. Organ. Psychol. 9, 621–640. 10.1017/iop.2016.6

[B26] Chan J. Wang J. (2018). Hiring preferences in online labor markets: evidence of a female hiring bias. Manage. Sci. 64, 2973–2994. 10.1287/mnsc.2017.275619642375

[B27] Cheryan S. Master A. Meltzoff A. N. (2015). Cultural stereotypes as gatekeepers: increasing girls' interest in computer science and engineering by diversifying stereotypes. Front. Psychol. 6:49. 10.3389/fpsyg.2015.00049 25717308 PMC4323745

[B28] Colquitt J. A. Rodell J. B. (2015). “Measuring justice and fairness,” in The Oxford Handbook of Justice in the Workplace, eds. R. S. Cropanzano & M. L. Ambrose (Oxford: Oxford University Press), 187–202.

[B29] Cummings M. L. (2006). Automation and accountability in decision support system interface design. J. Technol. Stud. 32, 23–31. 10.21061/jots.v32i1.a.4

[B30] Danks D. London A. J. (2017). “Algorithmic bias in autonomous systems,” in Proceedings of the Twenty-Sixth International Joint Conference on Artificial Intelligence, California. International Joint Conferences on Artificial Intelligence Organization, ed. C. Sierra (IJCAI). 10.24963/ijcai.2017/654

[B31] Daumeyer N. M. Onyeador I. N. Brown X. Richeson J. (2019). Consequences of Attributing Discrimination to Implicit vs. Explicit Bias. Charlottesville, VA: Center for Open Science. 10.31234/osf.io/42j7v

[B32] Davis A. Gentlewarrior S. (2015). White privilege and clinical social work practice: Reflections and recommendations. J. Progress. Hum. Serv., 26, 191–208. 10.1080/10428232.2015.106336137936550

[B33] Dietvorst B. J. Simmons J. P. Massey C. (2015). Algorithm aversion: people erroneously avoid algorithms after seeing them err. J. Exp. Psychol. Gen. 144, 114–126. 10.1037/xge0000033 25401381

[B34] Dovidio J. F. Gaertner S. L. (2000). Aversive racism and selection decisions: 1989 and 1999. Psychol. Sci. 11, 315–319. 10.1111/1467-9280.00262 11273391

[B35] Dovidio J. F. Gaertner S. L. (2010). “Intergroup bias,” in Handbook of Social Psychology, eds. S. T. Fiske, D. T. Gilbert, and G. Lindzey (Hoboken, NJ: Wiley), 1084–1121. 10.1002/9780470561119.socpsy002029

[B36] Endsley M. R. (2017). From here to autonomy. Hum. Factors 59, 5–27. 10.1177/0018720816681350 28146676

[B37] Enough B. Mussweiler T. (2001). Sentencing under uncertainty: anchoring effects in the courtroom1. J. Appl. Soc. Psychol. 31, 1535–1551. 10.1111/j.1559-1816.2001.tb02687.x

[B38] Eubanks V. (2019). Automating Inequality: How High-tech Tools Profile, Police, and Punish the Poor, First Picador Edition. New York, NY: Picador St. Martin's Press.

[B39] Faul F. Erdfelder E. Lang A.-G. Buchner A. (2007). G* power 3: a flexible statistical power analysis program for the social, behavioral, and biomedical sciences. Behav. Res. Methods 39, 175–191. 10.3758/BF03193146 17695343

[B40] Fejerskov A. M. (2021). Algorithmic bias and the (false) promise of numbers. Glob. Policy 12, 101–103. 10.1111/1758-5899.12915

[B41] Flage A. (2020). Discrimination against gays and lesbians in hiring decisions: a meta-analysis. Int. J. Manpow. 41, 671–691. 10.1108/IJM-08-2018-0239

[B42] Forret M. L. Turban D. B. (1996). Implications of the elaboration likelihood model for interviewer decision processes. J. Bus. Psychol. 10, 415–428. 10.1007/BF02251778

[B43] Gattino S. Miglietta A. Testa S. (2008). Dimensionality in Pettigrew and Meertens' blatant subtle prejudice scale. TPM. 15, 135–151.

[B44] Gawronski B. Bodenhausen G. V. (2006). Associative and propositional processes in evaluation: an integrative review of implicit and explicit attitude change. Psychol. Bull. 132, 692–731. 10.1037/0033-2909.132.5.692 16910748

[B45] Goddard K. Roudsari A. Wyatt J. C. (2012). Automation bias: a systematic review of frequency, effect mediators, and mitigators. J. Am. Med. Inform. Assoc. 19, 121–127. 10.1136/amiajnl-2011-000089 21685142 PMC3240751

[B46] Görlitz M. Gosten S. Schultz B. Rosenthal-von der Pütten A. (2023). “Teach me to be fair - an experimental study on fairness, self efficacy and human involvement in ADM systems,” in Proceedings of the 13th Conference of the Media Psychology Division (DGPs) (Esch-sur-Alzette: Melusina Press), 102.

[B47] Hyers L. L. (2007). Resisting prejudice every day: exploring women's assertive responses to anti-black racism, anti-semitism, heterosexism, and sexism. Sex Roles 56, 1–12. 10.1007/s11199-006-9142-8

[B48] Iyer A. Leach C. W. Crosby F. J. (2003). White guilt and racial compensation: the benefits and limits of self-focus. Pers. Soc. Psychol. Bull. 29, 117–129. 10.1177/0146167202238377 15272965

[B49] Jacowitz K. E. Kahneman D. (1995). Measures of anchoring in estimation tasks. Pers. Soc. Psychol. Bull. 21, 1161–1166. 10.1177/01461672952111004

[B50] Janssen M. Hartog M. Matheus R. Yi Ding A. Kuk G. (2022). Will algorithms blind people? The effect of explainable AI and decision-makers' experience on AI-supported decision-making in government. Soc. Sci. Comput. Rev. 40, 478–493. 10.1177/0894439320980118

[B51] Johnson G. M. (2021). Algorithmic bias: on the implicit biases of social technology. Synthese 198, 9941–9961. 10.1007/s11229-020-02696-y

[B52] Kaas L. Manger C. (2012). Ethnic discrimination in Germany's labour market: a field experiment. German Econ. Rev. 13, 1–20. 10.1111/j.1468-0475.2011.00538.x

[B53] Kahneman D. Tversky A. (1972). Subjective probability: a judgment of representativeness. Cogn. Psychol. 3, 430–454. 10.1016/0010-0285(72)90016-3

[B54] Koopmans R. Veit S. Yemane R. (2018). Ethnische Hierarchien in der Bewerberauswahl: ein Feldexperiment zu den Ursachen von Arbeitsmarktdiskriminierung, volume SP VI 2018-104 of Discussion Papers/Wissenschaftszentrum Berlin für Sozialforschung, Forschungsschwerpunkt Migration und Diversität, Abteilung Migration, Integration, Transnationalisierung. Berlin: Wissenschaftszentrum Berlin für Sozialforschung gGmbH.

[B55] Kordzadeh N. Ghasemaghaei M. (2022). Algorithmic bias: review, synthesis, and future research directions. Eur. J. Inform. Syst. 31, 388–409. 10.1080/0960085X.2021.1927212

[B56] Koriat A. Lichtenstein S. Fischhoff B. (1980). Reasons for confidence. J. Exp. Psychol. Hum. Learn. Mem. 6, 107–118. 10.1037//0278-7393.6.2.107

[B57] Krings F. Olivares J. (2007). At the doorstep to employment: discrimination against immigrants as a function of applicant ethnicity, job type, and raters' prejudice. Int. J. Psychol. 42, 406–417. 10.1080/00207590701251721

[B58] Kupfer C. Prassl R. Fleiß J. Malin C. Thalmann S. Kubicek B. (2023). Check the box! how to deal with automation bias in AI-based personnel selection. Front. Psychol. 14:1118723. 10.3389/fpsyg.2023.1118723 37089740 PMC10113449

[B59] Lacroux A. Martin-Lacroux C. (2022). Should i trust the artificial intelligence to recruit? recruiters' perceptions and behavior when faced with algorithm-based recommendation systems during resume screening. Front. Psychol. 13:895997. 10.3389/fpsyg.2022.895997 35874355 PMC9298741

[B60] Laux J. (2023). Institutionalised distrust and human oversight of artificial intelligence: towards a democratic design of AI governance under the European Union. AI Soc. 1–14. 10.1007/s00146-023-01777-z PMC11614927 39640298

[B61] Lee J. Moray N. (1992). Trust, control strategies and allocation of function in human-machine systems. Ergonomics 35, 1243–1270. 10.1080/00140139208967392 1516577

[B62] Lee M. K. Kim J. T. Lizarondo L. (2017). “A human-centered approach to algorithmic services: considerations for fair and motivating smart community service management that allocates donations to non-profit organizations,” in Proceedings of the 2017 CHI conference on human factors in computing systems (New York, NY), 3365–3376. 10.1145/3025453.3025884

[B63] Luong A. Kumar N. Lang K. R. (2021). Human-machine collaborative decision-making in organizations: examining the impact of algorithm prediction bias on decision bias and perceived fairness. Available at SSRN 3988456. 10.2139/ssrn.3988456

[B64] Lyell D. Coiera E. (2017). Automation bias and verification complexity: a systematic review. J. Am. Med. Inform. Assoc. 24, 423–431. 10.1093/jamia/ocw105 27516495 PMC7651899

[B65] Mahmud H. Islam A. N. Ahmed S. I. Smolander K. (2022). What influences algorithmic decision-making? A systematic literature review on algorithm aversion. Technol. Forecast. Soc. Change 175:121390. 10.1016/j.techfore.2021.121390

[B66] Manzey D. Reichenbach J. Onnasch L. (2012). Human performance consequences of automated decision aids: the impact of degree of automation and system experience. J. Cogn. Eng. Decis. Mak. 6, 57–87. 10.1177/1555343411433844

[B67] Marks M. J. Fraley R. C. (2006). Confirmation bias and the sexual double standard. Sex Roles, 54, 19–26. 10.1007/s11199-006-8866-9

[B68] Mosier K. L. Skitka L. J. Heers S. Burdick M. (2017). “Automation bias: decision making and performance in high-tech cockpits,” in Decision Making in Aviation, ed. D. Harris (London: Routledge), 271–288. 10.4324/9781315095080-16 11540946

[B69] Muir B. M. (1987). Trust between humans and machines, and the design of decision aids. Int. J. Man Mach. Stud. 27, 527–539. 10.1016/S0020-7373(87)80013-5

[B70] Oberst U. De Quintana M. Del Cerro S. Chamarro A. (2021). Recruiters prefer expert recommendations over digital hiring algorithm: a choice-based conjoint study in a pre-employment screening scenario. Manag. Res. Rev. 44, 625–641. 10.1108/MRR-06-2020-0356

[B71] O'Donnell R. M. (2019). Challenging racist predictive policing algorithms under the equal protection clause. NYUL Rev. 94:544.

[B72] Østerud K. L. (2023). Mental illness stigma and employer evaluation in hiring: Stereotypes, discrimination and the role of experience. Sociol. Health Illn. 45, 90–108. 10.1111/1467-9566.13544 36103320 PMC10087876

[B73] Parasuraman R. Riley V. (1997). Humans and automation: use, misuse, disuse, abuse. Hum. Factors 39, 230–253. 10.1518/00187209777854388618689046

[B74] Pettigrew T. F. Meertens R. W. (1995). Subtle and blatant prejudice in Western Europe. Eur. J. Soc. Psychol. 25, 57–75. 10.1002/ejsp.2420250106

[B75] Quillian L. and Midtbøen, A. H.. (2021). Comparative perspectives on racial discrimination in hiring: The rise of field experiments. Annu. Rev. Sociol. 47, 391–415. 10.1146/annurev-soc-090420-035144

[B76] Sánchez-Monedero J. Dencik L. Edwards L. (2020). “What does it mean to ‘solve' the problem of discrimination in hiring? social, technical and legal perspectives from the UK on automated hiring systems,” in Proceedings of the 2020 conference on fairness, accountability, and transparency (New YorkNY: ACM), 458–468. 10.1145/3351095.3372849

[B77] Sartori L. Bocca G. (2023). Minding the gap (s): public perceptions of AI and socio-technical imaginaries. AI Soc. 38, 443–458. 10.1007/s00146-022-01422-1

[B78] Schneider J. (2014). Diskriminierung am ausbildungsmarkt: Ausmaß, ursachen und handlungsperspektiven. Berlin: Forschungsbereich beim Sachverständigenrat deutscher Stiftungen für Integration und Migration (SVR).

[B79] Schuett J. (2023). Three lines of defense against risks from AI. AI Soc. 1–15. 10.1007/s00146-023-01811-034142373

[B80] Shafinah K. Selamat M. Abdullah R. Muhamad A. Noor A. (2010). System evaluation for a decision support system. Inf. Technol. J. 9, 889–898. 10.3923/itj.2010.889.898

[B81] Shaikh S. J. Cruz I. F. (2023). AI in human teams: effects on technology use, members' interactions, and creative performance under time scarcity. AI Soc. 38, 1587–1600. 10.1007/s00146-021-01335-5

[B82] Skitka L. J. Mosier K. Burdick M. D. (2000). Accountability and automation bias. Int. J. Hum. Comput. Stud. 52, 701–717. 10.1006/ijhc.1999.0349

[B83] Sloane M. Moss E. Chowdhury R. (2022). A silicon valley love triangle: Hiring algorithms, pseudo-science, and the quest for auditability. Patterns 3:100425. 10.1016/j.patter.2021.100425 35199067 PMC8848005

[B84] Sommers S. R. Norton M. I. (2006). Lay theories about white racists: what constitutes racism (and what doesn't). Group Process. Intergr. Relat. 9, 117–138. 10.1177/1368430206059881

[B85] Sosulski K. (2018). Data Visualization Made Simple: Insights into Becoming Visual. London: Routledge. 10.4324/9781315146096

[B86] Stamarski C. S. Son Hing L. S. (2015). Gender inequalities in the workplace: the effects of organizational structures, processes, practices, and decision makers' sexism. Front. Psychol. 6:1400. 10.3389/fpsyg.2015.01400 26441775 PMC4584998

[B87] Stewart L. D. Perlow R. (2001). Applicant race, job status, and racial attitude as predictors of employment discrimination. J. Bus. Psychol. 16, 259–275. 10.1023/A:1011113301301

[B88] Suen H.-Y. Hung K.-E. Lin C.-L. (2020). Intelligent video interview agent used to predict communication skill and perceived personality traits. Human-centric Comput. Inf. Sci. 10, 1–12. 10.1186/s13673-020-0208-3

[B89] Tal A. S. Batsuren K. Bogina V. Giunchiglia F. Hartman A. Loizou S. K.. (2019). ““End to end” towards a framework for reducing biases and promotingf transparency of algorithmic systems,” in *2019 14th International Workshop on Semantic and Social Media Adaptation and Personalization (SMAP)* (Larnaca: IEEE), 1–6. 10.1109/SMAP.2019.8864914

[B90] Tversky A. Kahneman D. (1974). Judgment under uncertainty: heuristics and biases: Biases in judgments reveal some heuristics of thinking under uncertainty. Science 185, 1124–1131. 10.1126/science.185.4157.1124 17835457

[B91] Vallone R. P. Ross L. Lepper M. R. (1985). The hostile media phenomenon: biased perception and perceptions of media bias in coverage of the Beirut massacre. J. Pers. Soc. Psychol. 49, 577–585. 10.1037//0022-3514.49.3.577 4045697

[B92] Van der Linden S. Panagopoulos C. Roozenbeek J. (2020). You are fake news: political bias in perceptions of fake news. Media Cult. Soc. 42, 460–470. 10.1177/0163443720906992

[B93] Varona D. Suarez J. L. (2023). Social context of the issue of discriminatory algorithmic decision-making systems. AI Soc. 1–13. 10.1007/s00146-023-01741-x37346393

[B94] Willemsen T. M. (2002). Gender typing of the successful manager—a stereotype reconsidered. Sex Roles 46, 385–391. 10.1023/A:1020409429645

[B95] Wood M. Hales J. Purdon S. Sejersen T. Hayllar O. (2009). “A test for racial discrimination in recruitment practice in British cities,” in Department for Work and Pensions Research Report 607, 1–69.

[B96] Yu K. Berkovsky S. Taib R. Conway D. Zhou J. Chen F.. (2017). “User trust dynamics: An investigation driven by differences in system performance,” in Proceedings of the 22nd international conference on intelligent user interfaces (New York, NY: ACM), 307–317. 10.1145/3025171.3025219

[B97] Zerilli J. Knott A. Maclaurin J. Gavaghan C. (2019). Algorithmic decision-making and the control problem. Minds Mach. 29, 555–578. 10.1007/s11023-019-09513-7

[B98] Zick A. Wolf C. Küpper B. Davidov E. Schmidt P. Heitmeyer W. (2008). The syndrome of group-focused enmity: The interrelation of prejudices tested with multiple cross-sectional and panel data. J. Soc. Issues 64, 363–383. 10.1111/j.1540-4560.2008.00566.x

